# Optimal Design of Steel–Concrete Composite Beams Strengthened under Load

**DOI:** 10.3390/ma14164715

**Published:** 2021-08-20

**Authors:** Piotr Szewczyk, Maciej Szumigała

**Affiliations:** 1 Faculty of Civil and Environmental Engineering, West Pomeranian University of Technology in Szczecin, Al. Piastów 17, 70-310 Szczecin, Poland; 2 Faculty of Civil and Transport Engineering, Poznan University of Technology, Ul. Piotrowo 3, 60-965 Poznań, Poland; maciej.szumigala@put.poznan.pl

**Keywords:** steel–concrete composite beams, strengthening, strain energy, optimization

## Abstract

This paper presents results of numerical analysis and experimental research on strengthening of steel–concrete composite beams. Studied members consisted of IPE200 I-beam and 90 × 700 mm reinforced concrete slab. The steel part of the section was strengthened by welding additional steel plates at the bottom. The study was performed for plate thickness ranging between 6 to 22 mm. Spatial FEM models were developed to account for material and geometric nonlinearities and for stress and post-welding strain. Proposed numerical models were experimentally validated. One aim was to find an optimum solution which would minimize cost and maximize bending capacity. To achieve this, energy parameters available in numerical simulations were reviewed and analyzed. Recoverable strain energy value determined in Abaqus was used to find the optimum solution.

## 1. Introduction

Composite structures, made by combining at least two materials with different properties, are designed with a view to making the best of both materials. The aim of design is to use their advantages. Although steel–concrete members are the most commonly used, other such as aluminum–concrete [1], aluminum–timber [2,3] and timber–concrete elements [4,5] can be applied. The paper analyzes steel–concrete beams typically considering concrete as operating under compression and steel I-beam operating under tension.

Composite structural members with extended service life often require repairs, modernization or strengthening. Technical expertise on the subject of strengthening typical steel and concrete structures is very extensive. We must not forget, however, about the limitations and difficulties posed by differences in material properties, e.g., brittle concrete or plastic steel. Composite elements, such as the ones included in this paper, can be reinforced in their concrete or steel parts. This paper discusses strengthening of the steel I-beams by welding additional metal plates to the bottom. Residual stress, including concrete and/or welding shrinkage, should be taken into account. It is also possible to use other popular composite materials [6,7]. However, this research focused on reinforcement with typical building materials.

The efficiency of the proposed solution should also be evaluated. It is easy to estimate the capacity of the strengthened materially heterogeneous element operating in the elastic range. However, this approach is often not economically viable and its efficiency depends on the structure’s capacity reserves before strengthening. Although analysis in the plastic range yields better efficiency, it requires more advanced tools. The study used the finite element method (FEM) implemented in Abaqus environment. The program enables simulation of nonlinear behavior of materials and structures. Welding process and concrete shrinkage can also be simulated.

Given the right calculation tool, optimization of the solution can be performed. The solution should meet the set requirements and ensure low costs. The total cost of strengthening should include material and labor costs. In case of welded steel plates, the smaller the element, the lower the welding costs. Therefore, the optimal solution is to choose the strengthening plate that will have the best cost/capacity increase ratio. Parametric analysis was conducted on how the size of steel plate used for strengthening affected the capacity of the strengthened beam. Energy parameters, determined with Abaqus, were also used. Eventually, recoverable strain energy was assumed to be the optimization criterion [8,9,10,11,12].

## 2. Research Models

**Figure 1:**
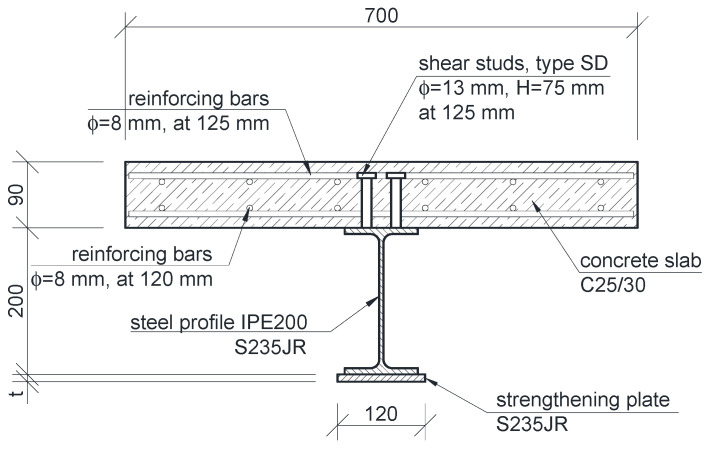
Cross-section of composite beam.

All beams analyzed in this study had the same cross-section. To fully exploit the properties of used materials, the neutral axis was designed close to the joint’s plane. Thus, the member was divided into compressed reinforced concrete and a steel I-beam in tension. The former was made of C25/30 concrete and BSt 500 reinforcement steel–dimensions given in Figure 1. The latter was IPE200 steel beam, made of S235JR steel. The joint was designed to be made of SD stud connectors whose spacing enabled full connection of the two parts of the section.

**Figure 2:**
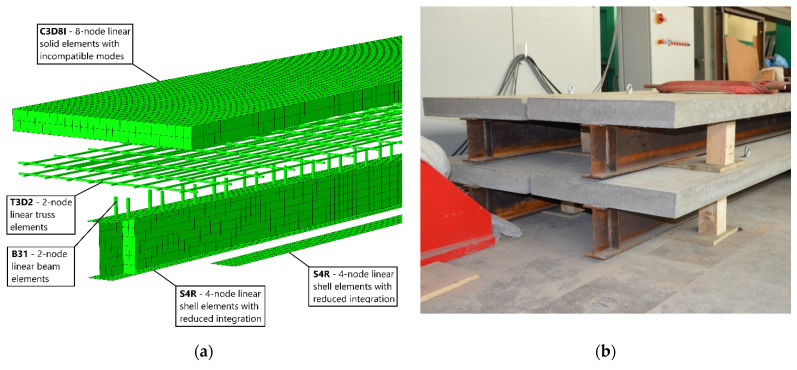
Models of composite beam: (**a**) numerical model and (**b**) experimental models.

Based on the above assumptions, numerical models were developed with Abaqus (Figure 2a) and physical experimental models were prepared (Figure 2b).

**Figure 3:**
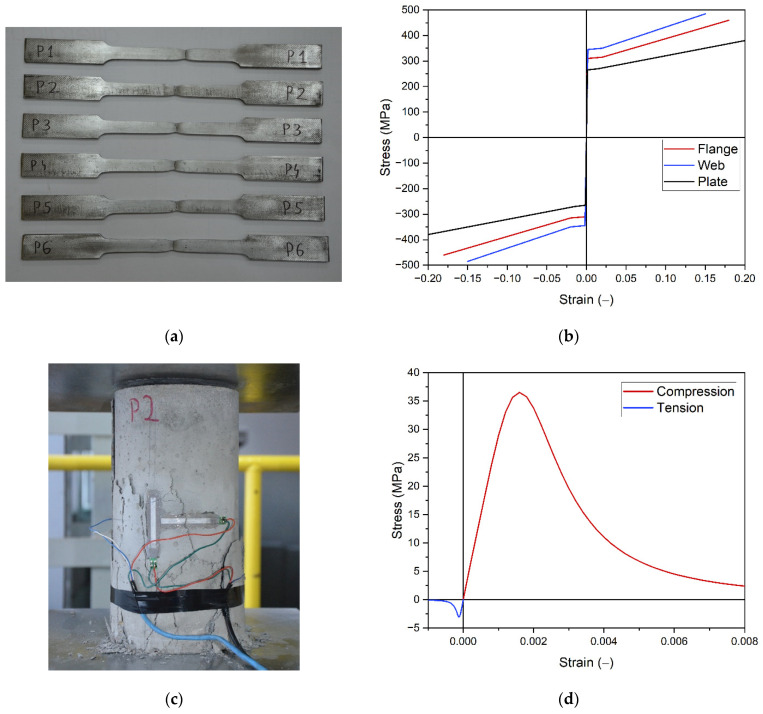
Constitutive laws of materials: (**a**,**c**) selected material samples used to define constitutive laws; (**b**) steel models; (**d**) concrete model.

The numerical models simulate both elastic and plastic operation of materials. The following constitutive laws were used: steel (Figure 3b) was modeled as an elastic–plastic material with yield point and strain hardening [13,14]; concrete (Figure 3d) was defined using Concrete Damage Plasticity model [15,16,17,18,19,20]. The model can independently describe the behavior of concrete under compression and tension. It can also account for stiffness degradation. The material parameters were in experimental tests. The view of selected samples is presented in Figure 3a,c. Steel properties were determined in a tensile test (separately for the flange, web and strengthening plate). A compressive strength, flexural strength and tensile splitting strength test were conducted to determine the properties of concrete.

**Figure 4:**
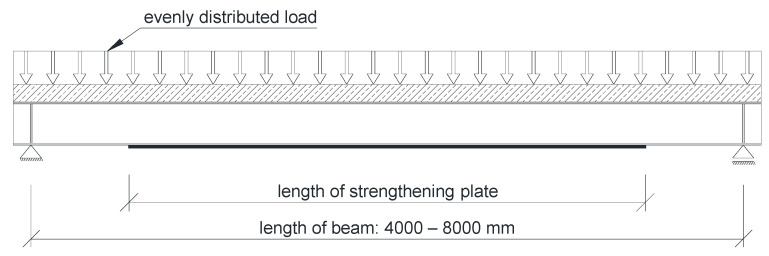
Static scheme of the analyzed beams.

All beams were analyzed in a simply supported system (Figure 4). Five beams of different length were tested. Support span varied between 4 and 8 m, with 1 m spacing. The beams were strengthened by welding plated to the bottom flange (Figure 1 and Figure 4). The length of the strengthening plate was one of the variables investigated in the parametric analysis, as shown in Section 3.

**Figure 5:**
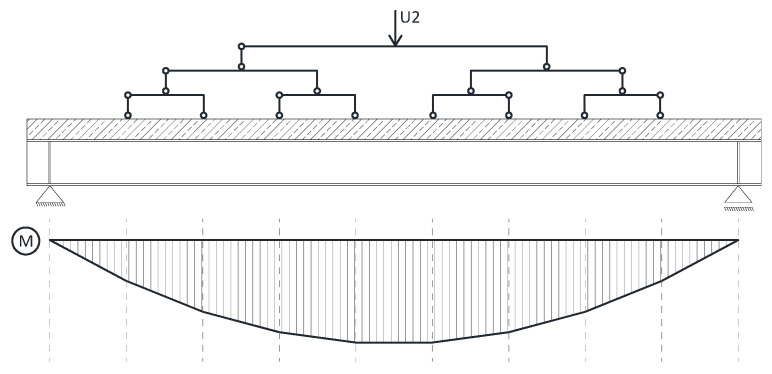
Schematic diagram of an evenly distributed load introduced with displacement.

An evenly distributed load acting on the reinforced concrete slab was applied to all models. The load was continuously applied till the failure of the element. To be able to observe decrease of stiffness and the failure moment, the load was applied using set displacement rate. Unfortunately, as Abaqus lacks this functionality, it was necessary to introduce a solution that would simulate the distributed load with simultaneous load application through displacement. Figure 5 shows a scheme of the applied solution. The loading mechanism allowed to control the U2 displacement at the top of a “pyramid’ that consisted of a number of statically determined flat frames. The load is distributed into 8 equal forces that simulate evenly distributed loads. Thanks to this solution the resulting distribution of bending moments is close to a parabola.

## 3. Numerical Analysis

### 3.1. Introduction

The fundamental assumption in this study was that the strengthening plate would be added for an element that is already operating under load [21,22,23,24,25]. Typically, structural members are operating under their dead load, a load from finishing layers, and live loads. From the numerical point of view, a change in the model parameters (addition of the strengthening plate) must be made during the simulation. The Abaqus software enables such analysis without interrupting the calculations. In each model, two modes of operation can be distinguished: before and after strengthening. The moment of strengthening can be very well observed in static equilibrium paths of such elements. Force–displacement paths were used in the study. Force was defined as the sum of total load acting on the beam. The displacement was measured in the middle of beam’s span.

**Figure 6:**
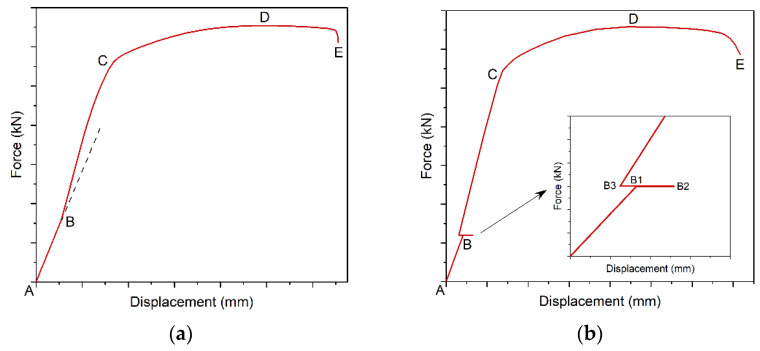
Static equilibrium path of strengthened beam: (**a**) without welding distortions and (**b**) with welding distortions.

Figure 6a shows an example static path. Line segment AB describes a beam without strengthening. Strengthening occurs in point B. Since after strengthening, the section has a higher stiffness, applied load causes smaller displacements. The equilibrium path shows this as a change of inclination angle of the curve relative to the OX axis. Figure 6a shows the tangent to the initial direction of static equilibrium path, which illustrates changed stiffness of the beam after strengthening. Line BC shows the beam’s operation in the elastic range. If load is increased the beam starts to operate in the plastic range (CD) and finally is damaged (DE).

Beam extension can bring about additional effects, such as stress and post welding strain [26,27,28,29,30]—Figure 6b. Welding begins in point B1. When the bottom part of the section heats up due to welding, deflection increases up to B2. Once welding is over, shrinkage occurs which reverses displacement direction. B3 shows the beam after cooling down. As the load is constant, only displacement changes. A separate analysis was made on the welding process and its effects [31]. This paper does not account for these impacts as they had a negligible effect on parameters used to find the optimum solution.

**Figure 7:**
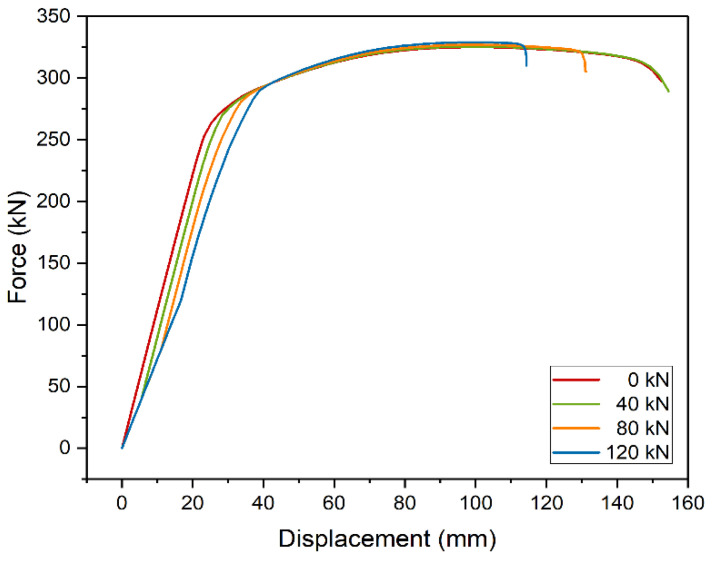
Static equilibrium paths of strengthened beams at different initial load levels.

First, the effect of initial load on the shape of the static equilibrium paths was analyzed (Figure 7). The calculations were made for the model of a 5 m beam strengthened with metal plate, 10 mm thick and 3200 mm long. Four load levels were considered: 40 kN (~75 MPa), 80 kN (~150 MPa), 120 kN (~225 MPa), and no load. The brackets contain the values of stresses in the steel beam in the place where welding was performed. In plastic analysis, the level of stresses during strengthening does not affect the final capacity. This effect is due to stress redistribution between the strengthened and strengthening parts. Interestingly, models strengthened at higher initial stress levels were less prone to plastic deformation and were damaged at lower displacement. In terms of safety, it is an adverse effect.

**Figure 8:**
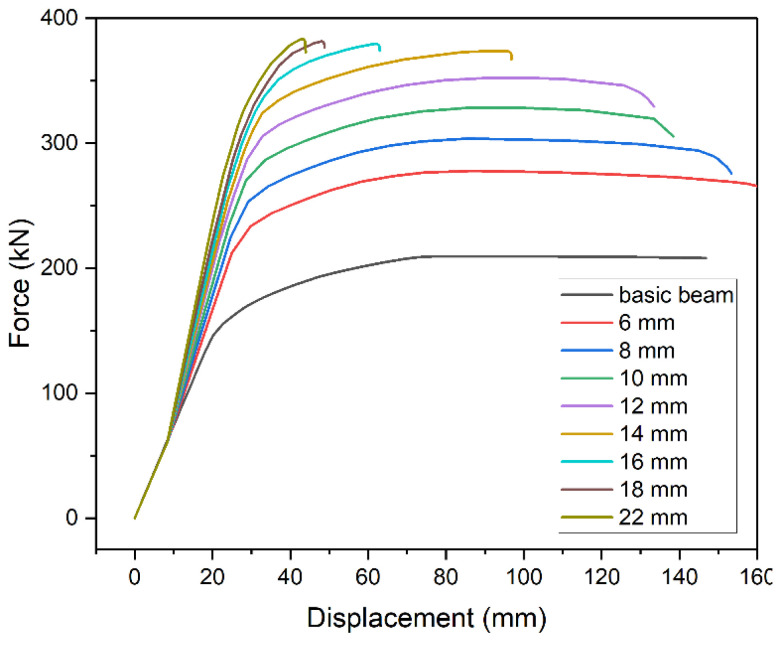
Static equilibrium paths of strengthened beams at different thickness of the plate.

Next, the effect of metal plate thickness was analyzed. The 5 m beam model was strengthened with metal plate 3500 mm long. The thickness varied between 6 and 22 mm (Figure 8). Thicker metal plate inevitably increases beam’s capacity. However, the increase is nonlinear and has a limited range in terms of thickness. Excessive thickness shortens the plastic part of static equilibrium path and thus renders plastic stress redistribution impossible. In our case, use of plates with thickness of over 14 mm did not significantly increase the load-bearing capacity however noticeably decreasing the safe displacement range.

**Figure 9:**
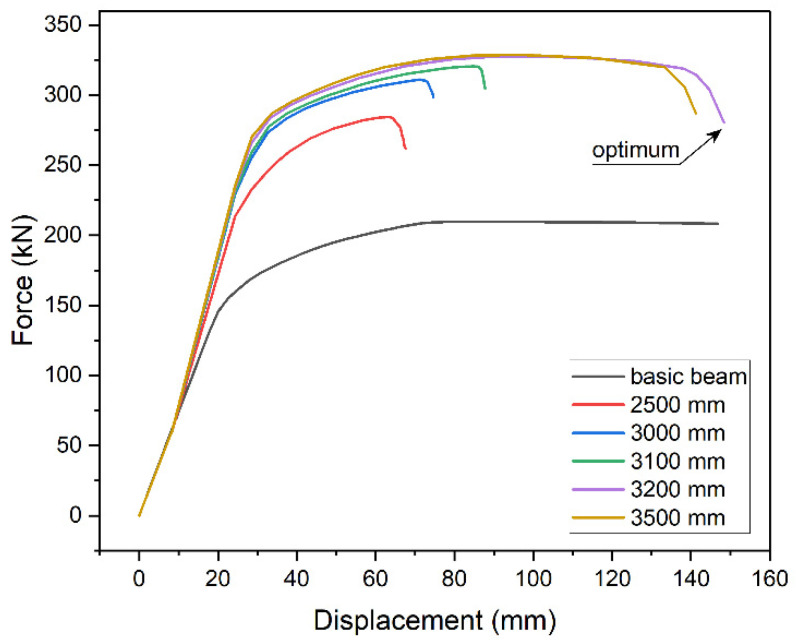
Static equilibrium paths of strengthened beams at different lengths of the plate.

The above effect results from the need to use longer and longer plates with the increase of their thickness. The effect of the length of strengthening plate on the beam’s capacity was shown with the 5 m beam model, strengthened with 10 mm plate of varied length. Figure 9 shows examples of static equilibrium paths. When the plate is too short, capacity decreases and the plastic part of equilibrium path shortens. The same effect is presented in Figure 8 for plates thickness over 16 mm. Increasing the length of the plate improves the load-bearing capacity, up to the point of plastic stress distribution in the section, and extends yield point, which is a noticeable advantage.

**Figure 10:**
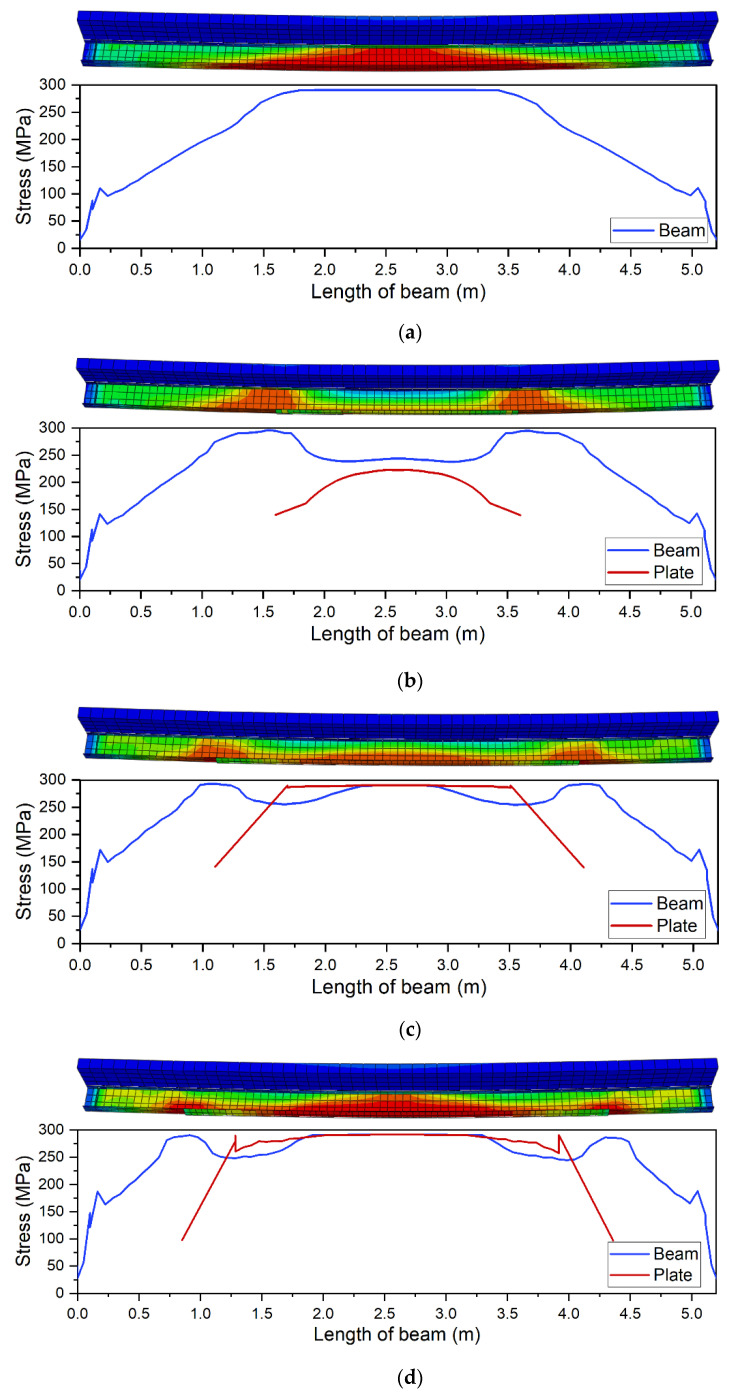
Distribution of normal stresses in the bottom flange of the steel beam and the strengthening plate: (**a**) beam without strengthening; (**b**) beam strengthened with a 2000 mm plate; (**c**) beam strengthened with a 3000 mm plate; (**d**) beam strengthened with a 3200 mm plate (optimum).

The effect is also presented in Figure 10 that shows von Mises stresses in the bottom flange (blue line) and in the strengthening plate (red line). Figure 10a shows an example of stress distribution in a beam without strengthening with highlighted yield zone. The introduction of strengthening visibly changes this distribution. Stress concentrates at the point of sudden stiffness change. Figure 10b shows a case when the applied plate was to short (2000 mm). Plastic deformation can only be observed at the place where the structural notch occurs, i.e., at the end of the strengthening plate. Stress in the strengthening plate does not even reach the yield point, rendering stress redistribution impossible. Figure 10c shows a case where longer plate was used (3000 mm). Although stress in the strengthening plate reaches yield point, the plastic zone is too small to enable full stress redistribution. The use of optimum length (3200 mm) enables full stress redistribution and provides the effective use of the added material (Figure 10d). Further increase of the length is not beneficial. On the contrary, it slightly shortens yield point (Figure 9).

### 3.2. Finding Optimum

Analysis of diagrams presented in Section 3.1 shows that there exists a certain optimum that ensures maximum capacity and high plastic deformation potential with minimum size of strengthening plate.

Parametric analysis was conducted to find the strengthening plate, with s_1_ length, s_2_ width and s_3_ thickness. The aim was to find design vector s = [s_1_, s_2_, s_3_], which would have the lowest required volume V = s_1_∙s_2_∙s_3_ (the volume of material can be used as an estimate of strengthening cost) and meet following requirements:

0 < s_1_ < L,
s_2_ = b_f_ + 20 mm,
6 mm < s_3_ < 30 mm, (1)

Length s_1_ is within the spacing of beam supports. Width s_2_ was assumed to be constant. It results from the width of bottom flange b_f_ extended on both sides by 10 mm, which enables connection of elements by fillet welds, applied in a horizontal position (PB). The limitation of thickness s_3_ is due to technology of fillet welding. Design variable s_3_ is a discrete variable of plate thickness, with the interval of 2 mm.

Energy parameters were used to find the optimum solution [32,33]. Abaqus can be used to determine 18 different energy parameters for each calculation increment. Since the discussed problem was static, some parameters equaled zero. Strain-related energy parameters had values other than zero:

- Recoverable strain energy (ALLSE) is related to elastic strain. It equals the area under the static equilibrium path in the elastic range. It is recoverable, i.e., it is spent when the load is removed and the model goes back to its initial shape. For an undeformed body, it equals zero.
- Energy dissipated by plastic deformation (ALLPD) is dissipated through permanent deformation, which remain permanent even in unloaded conditions.
- Energy dissipated by damage (ALLDMD) is related to damage occurring in the construction. In the analyzed case, it was approximately one thousand times smaller than the energies mentioned above. Its increase correlates with crack propagation in concrete. It occurs in the descending part of static equilibrium path. For these reasons, it was omitted in analysis.
- Total strain energy (ALLIE). It is the sum of ALLSE, ALLPD, and ALLDMD. The latter has a negligible effect on ALLIE.

**Figure 11:**
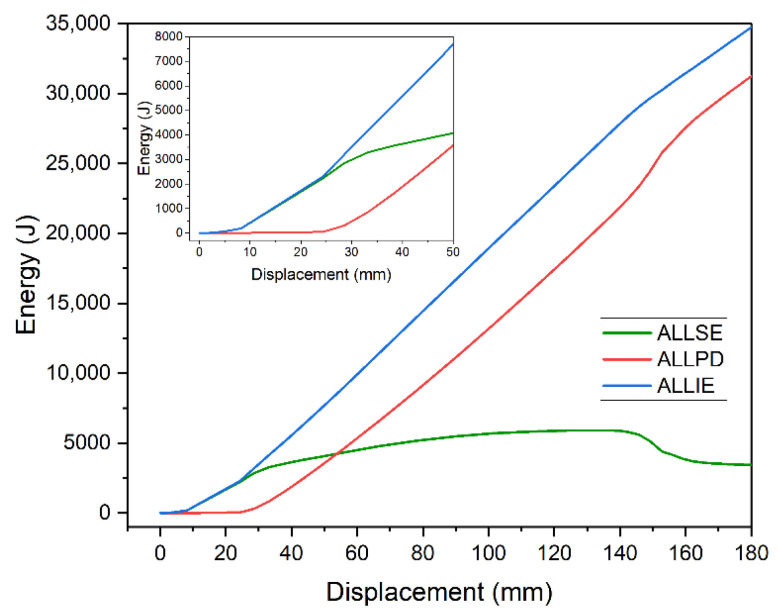
Change of energy value as a function of beam displacement.

Figure 11 shows changes of energies listed above for the given model—a 5 m beam strengthened with plate 10 mm thick and 3200 mm long. As long as the beam operated in the elastic range (deflection of 24 mm), recoverable strain energy ALLSE equaled total strain energy ALLIE. At the same time, energy dissipated by plastic deformation (ALLPD) was zero. ALLPD constantly increased as plastic zone developed. For 145–155 mm deflection, energy behaved in an interesting way. ALLSE noticeably decreased and ALLPD increased. At this point, the static equilibrium path suddenly goes down. This point is tantamount to fracture of the element.

**Figure 12:**
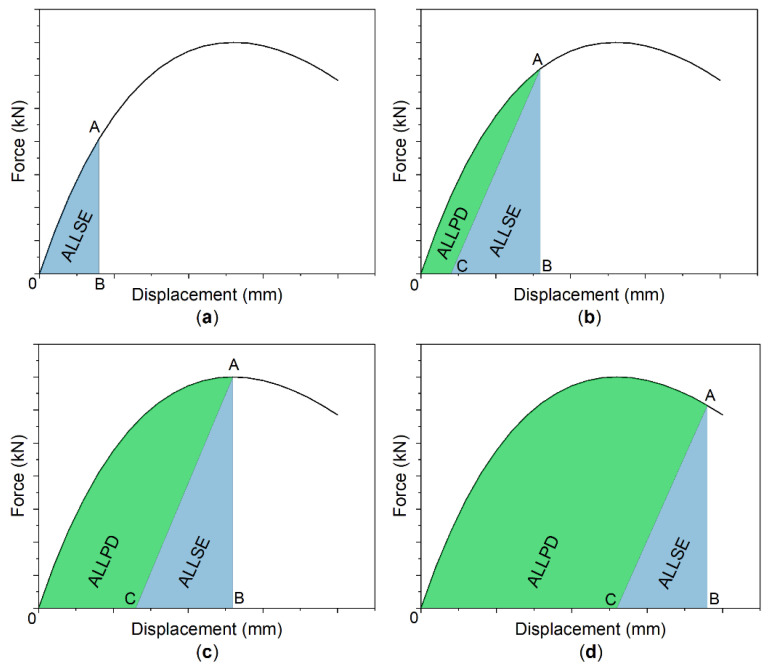
ALLSE and ALLPD energy definition: (**a**) elastic range and (**b**,**c**,**d**) plastic range.

This effect is presented in Figure 12. In the elastic range (Figure 12a), ALLSE equals ALLIE, which is determined by the area of triangle OAB. When the beam enters into the plastic range (Figure 12b), permanent deformation occurs where ALLPD is dissipated. This energy can be defined by the area of triangle OAC. When it reaches beam’s capacity (Figure 12c), the area of triangle CAB is the biggest. Therefore, ALLSE reaches maximum. Once capacity limit is exceeded (Figure 12d), ALLSE drops and is replaced by ALLPD.

**Figure 13:**
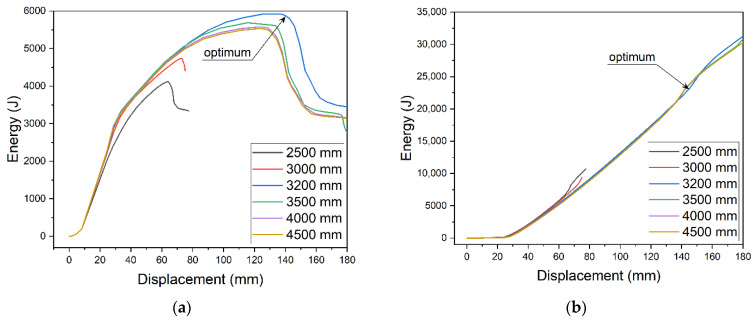
Energy value depending on the length of the strengthening plate: (**a**)ALLSE and (**b**) ALLPD.

When the strengthening plate dimensions change, energy parameters change as well. Energy changes were analyzed for the 5 m beam model with 10 mm thick strengthening plate. The determined optimum length of the plate was 3200 mm. For that length (Figure 13a), ALLSE reaches its maximum at the maximum displacement before failure. At the same time, ALLPD increase (Figure 13b) occurs at the maximum displacement.

Recoverable strain energy ALLSE used as the optimization criterion allows to find a solution with the highest capacity. The solution is characterized by the highest strain ability before damage. Its static equilibrium path has two important properties. First, it achieves capacity resulting from plastic stress redistribution between the strengthened and strengthening elements. Second, it has the longest part before the inflection point, i.e., failure occurs at the maximum displacement.

**Figure 14:**
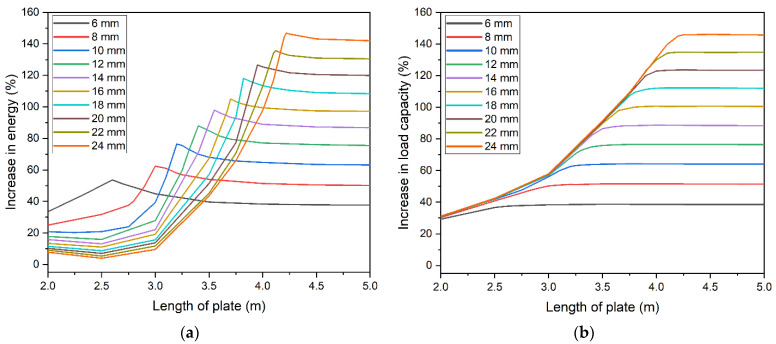
Parametric analysis of strengthening plate dimension: (**a**) influence on recoverable strain energy ALLSE and (**b**) influence on load capacity.

Based on the above assumption, a number of parametric analyses were conducted to determine the effect of plate size on ALLSE. Increase in load capacity was determined for all studied cases. For each thickness, models were developed with varied length—40–100% of the beam’s length. The percentage increase of ALLSE and capacity in relation to the model without strengthening was determined. Results for a 5 m beam are presented in Figure 14. Figure 14a shows ALLSE changes for each thickness of the strengthening plate. The extrema of the function change with change of the thickness. For thicker plates, the extrema become less indistinguishable. When thickness exceeded 24 mm, they could not be determined. Figure 14b shows capacity changes. A similar trend can be observed. In order for thicker plate to achieve maximum capacity, their length must be increased.

**Figure 15:**
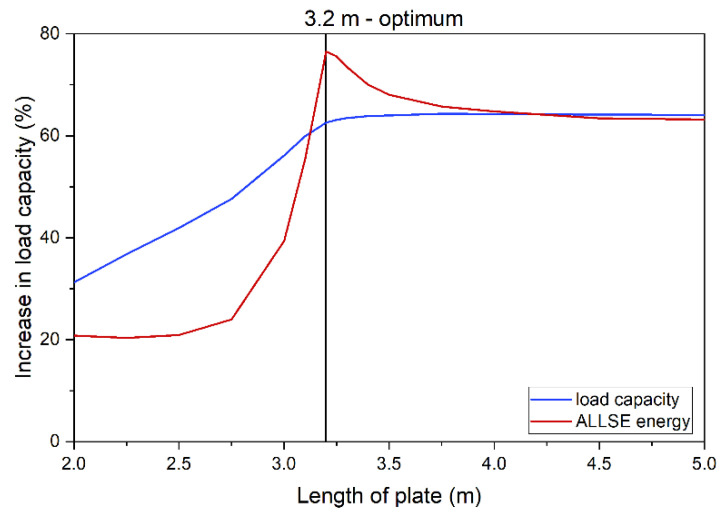
Comparison of energy and load capacity variability for a 5 m beam.

A comparison of ALLSE and capacity changes shows that energy maximum overlaps with the beam reaching its maximum capacity (Figure 15). This concurs with the assumption that the energy criterion can be used to find the optimum solution. Figure 15 shows a beam strengthened with a 10 mm thick plate. In this case, the optimum length is 3200 mm. Figure 9 confirms this and shows that this model has the longest static equilibrium path. Additionally, Figure 10d shows that the model enables full yield and redistribution of stress between the bottom flange and the strengthening plate.

**Figure 16:**
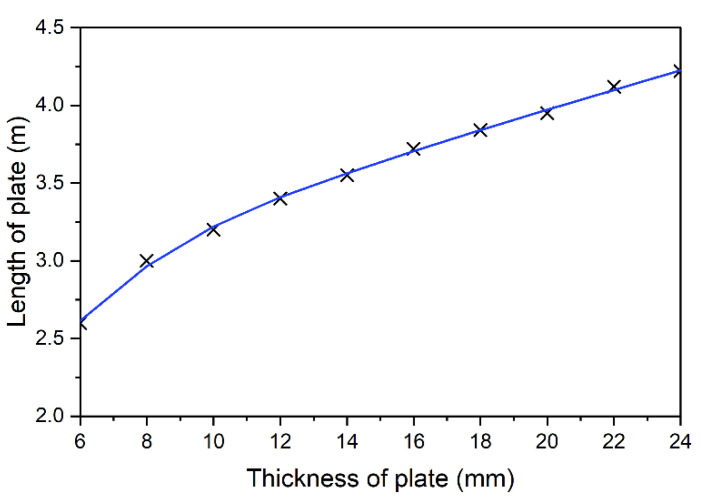
Optimal solutions for a 5 m beam.

Based on that the results, a curve was drawn to describe the optimum selection of plate length in relation to its thickness, Figure 16. For thinner strengthening, the relation is nonlinear.

**Figure 17:**
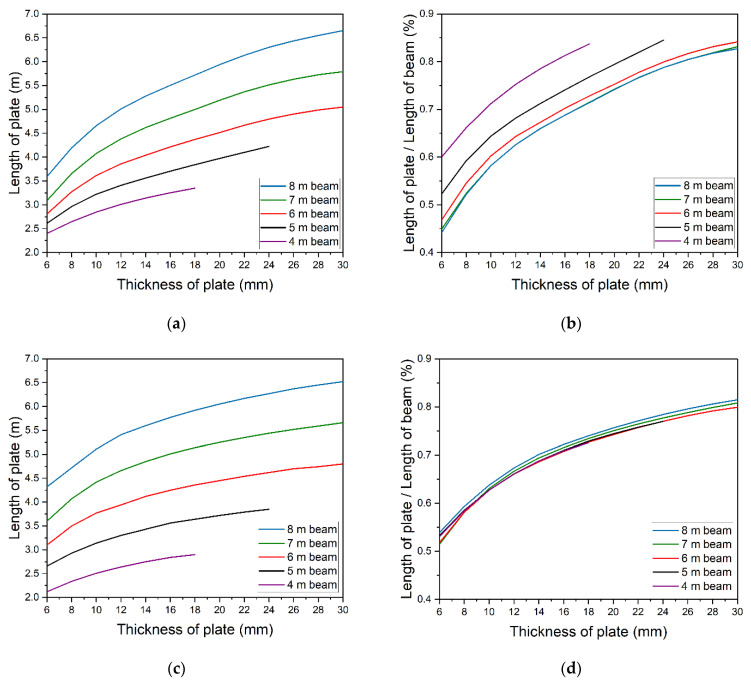
Optimal solutions for beams 4–8 m long: (**a**,**b**) determined by the energy method and (**c**,**d**) defined by the envelope of moments.

The same parametric analysis was also conducted for different beams of the same cross-section and length of 4, 6, 7, and 8 m. Results are presented in Figure 17a. The lack of energy extrema for 4 m and 5 m beams made it impossible to determine the optimal thickness of the plate. Figure 17a clearly shows that longer beams require longer strengthening plates. The dependency is nonlinear. Figure 17b shows the relation between the optimum length of strengthening plate and beam’s length. Relatively longer plates must be used to strengthen shorter beams. As the beam’s length increases, the required length of strengthening drops and finally stabilizes at a constant level. The solutions for 7 and 8 m beams are similar and are only slightly different from the optimum solution for strengthening the 6 m beam.

**Figure 18:**
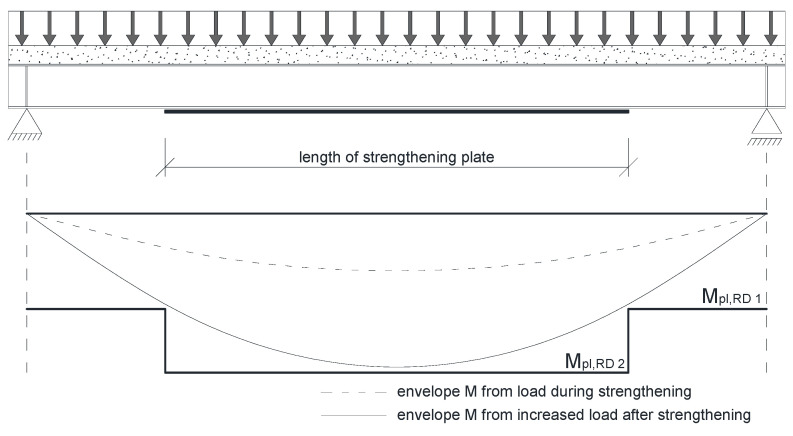
Envelope of moments in a strengthened beam.

The required length of the plate can also be found in a simple, engineering way by developing an envelope of bending moments. An example of that is shown in Figure 18. The M_pl,RD1_ and M_pl,RD2_ are section capacity before and after strengthening, respectively. The area when beam’s capacity exceeds the envelope shows the strengthening degree. In this way, correct lengths of strengthening were determined for all models (Figure 17c). In this case, the beam’s length does not affect the solution as all results are based on geometric data (Figure 17d).

**Figure 19:**
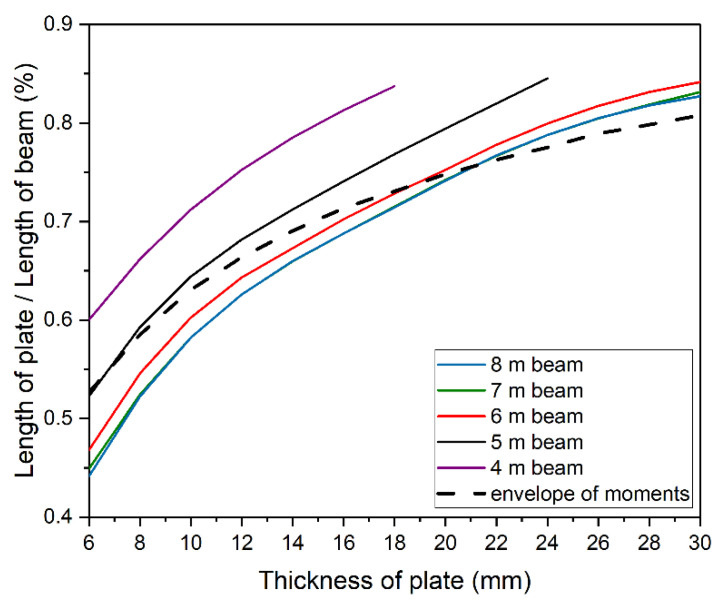
Comparison of optimal solutions defined by the energy method and the envelope of moments.

Figure 19 compares energy criterion related optimum solutions from Figure 17b (color solid lines) with those of bending moment envelope from Figure 17d (black dashed line). Bending moment envelope results concur with numerical results for longer beams, 6 ÷ 8 m long. For thinner plates, numerical calculation allows the use of plates shorter than indicated by envelope. Results are slightly overestimated for thicker plates.

Longer plates in relation to beam’s length are required to reach the optimum for the shortest beams. Evidently, more factors should be accounted for when determining capacity, including the effect of shear force which is particularly significant for shorter beams.

**Figure 20:**
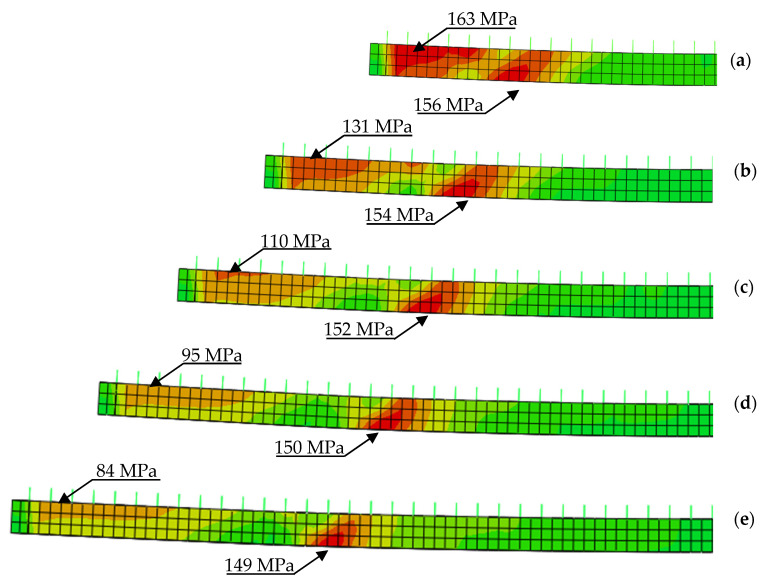
Tangential stresses in the web of a steel beam: (**a**) 4 m beam; (**b**) 5 m beam; (**c**) 6 m beam; (**d**) 7 m beam; (**e**) 8 m beam.

**Figure 21:**
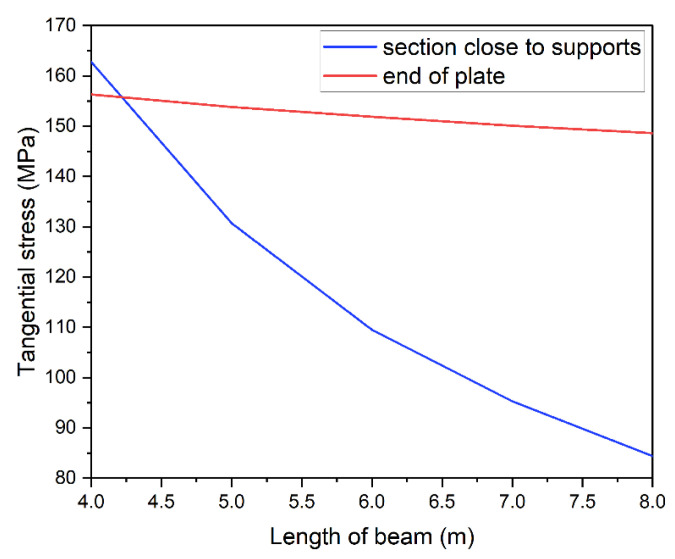
Maximum tangential stresses depending on the length of the beam.

Tangential stress distribution was analyzed in the steel section. The introduction of strengthening plate changes the normal and tangential stress distribution. Figure 20 shows tangential stress distribution in the web of the I-beam. Curves were made for the optimum models of all beam’s lengths strengthened with 10 mm thick plate. Every figure shows a clear concentration of tangential stress near the plate ends. Regardless of beam’s length the value of stresses reached approximately 150 MPa (red line in Figure 21). Given the limit value of tangential stress for S235JR steel, obtained results are considered as dangerously high. This is due to the fact that the model includes real yield point for the used material. Visible changes of tangential stress can be observed in sections close to supports. They seem to correlate with beam’s length. The shorter the beam, the higher the tangential stress exceeding in some cases 160 MPa.

This dependence is presented in Figure 21 with a blue line. Its shape is similar to curves in Figure 17, i.e., higher stress in the support zone is related to use of longer strengthening plates.

## 4. Experimental Verification

**Figure 22:**
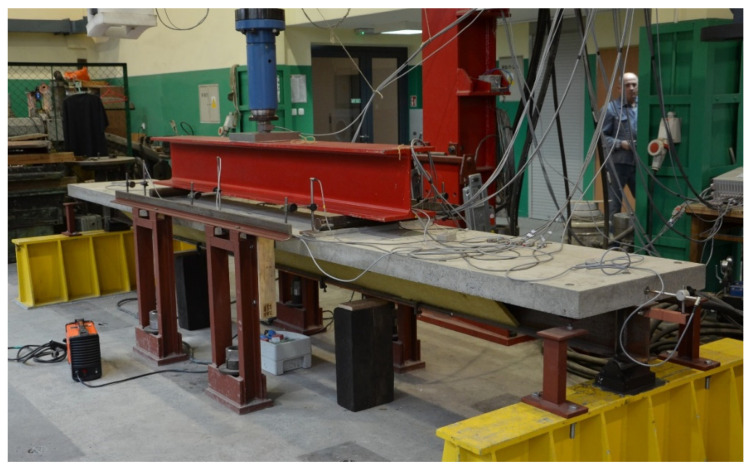
One of the beams on the test stand.

**Figure 23:**
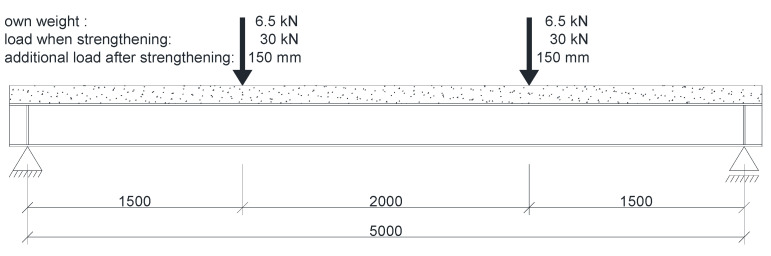
Location and values of loads acting on the test beams.

To check whether or not numerical models were correct, they were experimentally verified. Three identical beams with a section shown in Figure 1 were prepared and tested [34]. Their total length of 5200 mm allowed the spacing between supports to be 5000 mm (Figure 22 and Figure 23).

Numerical analysis was conducted for models with uniformly distributed load, which is difficult to achieve experimentally. Therefore, two points of load applied symmetrically were used, with a spacing of 2000 mm (Figure 23).

**Figure 24:**
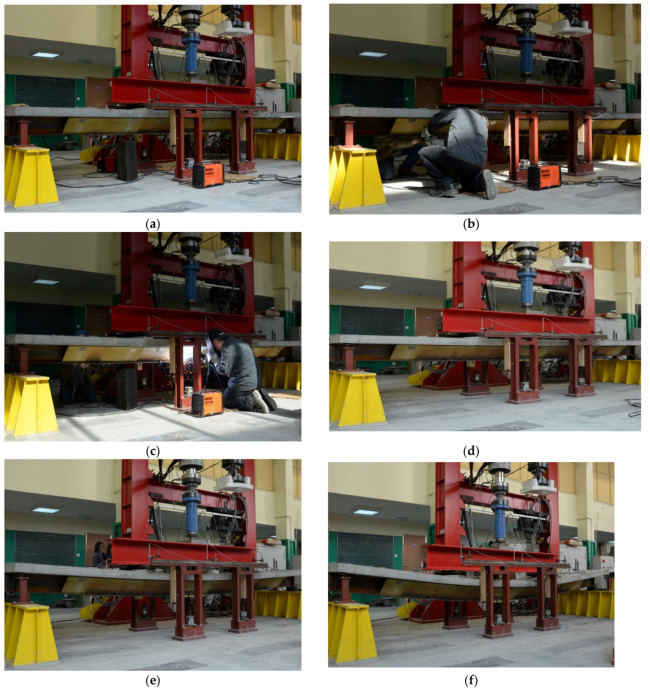
The main stages of experimental research: (**a**) initial state—before the beginning of the research; (**b**) introduction of the constant load and assembly of the strengthening plate; (**c**) welding of a strengthening plate; (**d**) cooling down after welding; (**e**) increasing the load after strengthening; (**f**) beam destruction.

Figure 24 shows the main stages of experimental research. Figure 24a shows the state before the test, when only the initial load is acting on the beam. The initial load was defined as the model’s own weight plus weight of intermediate beam resting on it (HEB320) used to transfer load from a hydraulic actuator on to the model. The total mass of the model and the intermediate beam imposed a load of 13 kN. Then, the load acting on the structure during the strengthening process was introduced. The constant load of 60 kN caused 163 MPa stress in the welding point. Load was controlled with force in the experiment and in the numerical model. It enabled recording of displacement caused by welding. At this point, the strengthening plates were installed (Figure 24b). The beams were strengthened with 10 mm thick plate, which increased capacity by approximately 50%. A plate with a length of 3300 mm was determined with the energy criterion. Figure 24c shows the process of welding of the strengthening plate to the bottom flange of the I-beam. After finishing welding, the structure was allowed to cool down completely (Figure 24d). At that time, the welding shrinkage of welds located below the neutral axis reduced the deflection value of the beam. Then, the load acting on the beam was increased (Figure 24e). The application of a displacement of 150 mm resulted in the final failure of the beams (Figure 24f).

**Figure 25:**
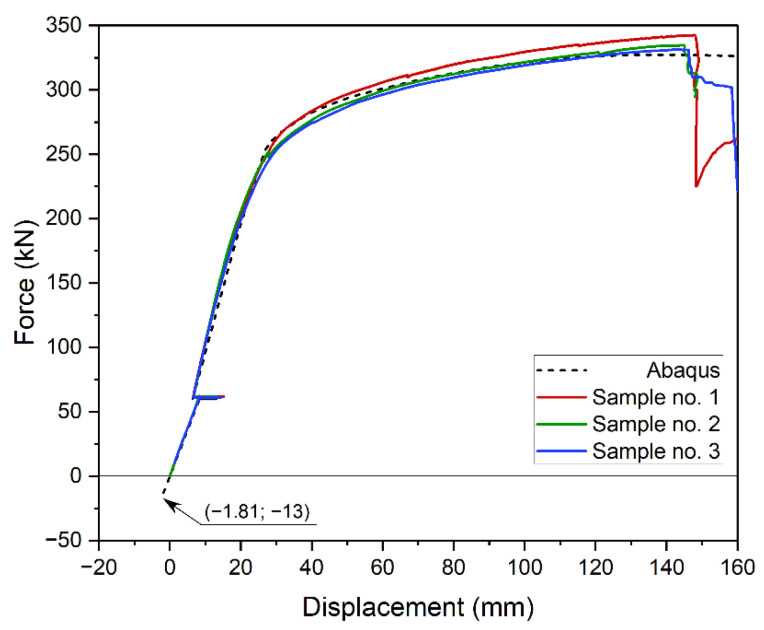
Comparison of experimentally and numerically obtained static equilibrium path.

Figure 25 shows experimental (continuous line) and numerical (dotted line) static equilibrium paths. In the experiment, force was read from a load cell between the beam and the actuator. It was numerically determined as a sum of vertical reactions on the supports. Displacement is defined as beam deflection in the middle of its span. It was experimentally read with inductive transducers (LVDT). Numerically, it is displacement of a selected node of finite element mesh.

When comparing obtained results, it is important to account for the structure’s own weight. Therefore, the beginning of coordinate system was defined as the start of the experiment. The numerical result was adjusted by 13 kN and relative deflection of 1.81 mm. Results are compared in Figure 25. Bearing in mind that the tested element was partly made of brittle material (concrete), the convergence of result is satisfactory. The maximum error between experimental and numerical average beam’s capacity is approximately 2.5%. Larger discrepancies appeared at yield point and crack propagation in concrete. The end of beam’s operation was slightly underestimated in the model. It could have been due to density differences between concrete samples used to define the model and concrete used in the slab.

## 5. Discussion and Conclusions

Currently, on the one hand, we are dealing with a rapid development of civil engineering, but on the other hand, we are facing the vision of ending natural resources. In this situation, rational resource management becomes very important. Therefore, modernizations, extensions, and changes in the use of existing buildings are becoming more and more common. The issue of strengthening buildings is fully in line with the trend of sustainable development. This work presents a selected aspect of strengthening steel and concrete composite structures with the use of modern calculation tools.

An ideal tool for advanced structural analysis is the Finite Element Method (FEM). The paper presented a numerical FEM model of steel–concrete composite beam. This 3D model accounts for material, geometric nonlinearity and residual stress caused by welding. An algorithm modeling the successive stages of strengthening the structure under load was also developed. In the case of numerical analyzes themselves, the question of their compliance with real-life specimen remains. Therefore, the model has been successfully validated in experimental studies.

The strengthening of the composite beam was realized by welding a steel plate to the lower flange of the I-beam. A series of parametric analyses was conducted using the model. The analyzed problems included: the effect of strengthening plate on capacity, the ability of composite beams to undergo plastic strain. Energy analysis was used to find the optimum solution. Strain—related energy changes were investigated. Energy resulting from elastic and plastic strain was considered. The maximum recoverable strain energy (ALLSE) was defined as the optimization criterion. That was useful to find a solution that allowed full stress redistribution between the strengthened and strengthening parts, using the smallest amount of plate. Minimizing the dimensions of the strengthening plates reduces costs. At the same time, it leads to a reduction in the emission of carbon dioxide generated at the production stage, a reduction in the energy needed for welding and a reduction in the emission of welding gases and dust.

The comparison of results produced with recoverable strain energy (ALLSE) and those generated with bending moment envelope brings interesting results. ALLSE—based solutions can account for additional factors that affect the structure, e.g., the effect of shear force.

Results presented in the study confirm the contention that energy analysis can be used as an effective tool to find the optimum solution. The tool proved to be useful even when solving a complex nonlinear task. It also made it possible to simulate the strengthening under load by changing the geometry of the model during the calculations.

## Data Availability

Data sharing is not applicable to this article.

## References

[1] Polus Ł., Szumigała M. (2019). An experimental and numerical study of aluminium–concrete joints and composite beams. Arch. Civ. Mech. Eng..

[2] Chybiński M., Polus Ł. (2019). Theoretical, experimental and numerical study of aluminium-timber composite beams with screwed connections. Constr. Build. Mater..

[3] Saleh S.M., Jasim N.A. (2014). Structural Behavior of Timber Aluminum Composite Beams under Static Loads. IJERT Int. J. Eng. Res. Technol..

[4] Szumigała M., Szumigała E., Polus Ł. (2018). Laboratory tests of new connectors for timber-concrete composite structures. Eng. Trans..

[5] Hadigheh S.A., McDougall R., Wiseman C., Reid L. (2021). Evaluation of composite action in cross laminated timber-concrete composite beams with CFRP reinforcing bar and plate connectors using Digital Image Correlation (DIC). Eng. Struct..

[6] Mercedes L., Escrig C., Bernat-Masó E., Gil L. (2021). Analytical approach and numerical simulation of reinforced concrete beams strengthened with different frcm systems. Materials.

[7] Subhani M., Kabir M.I., Al-Ameri R. (2020). Strengthening of steel-concrete composite beams with composite slab. Steel Compos. Struct..

[8] Simonetti H.L., de Assis das Neves F., Almeida V.S. (2021). Multiobjective topology optimization with stress and strain energy criteria using the SESO method and a Multicriteria Tournament Decision. Structures.

[9] Bagherinejad M.H., Haghollahi A. (2020). Study on Topology Optimization of Perforated Steel Plate Shear Walls in Moment Frame Based on Strain Energy. Int. J. Steel Struct..

[10] Huang M., Lei Y. (2020). Bearing Damage Detection of a Reinforced Concrete Plate Based on Sensitivity Analysis and Chaotic Moth-Flame-Invasive Weed Optimization. Sensors.

[11] Liu B., Guo D., Jiang C., Li G., Huang X. (2019). Stress optimization of smooth continuum structures based on the distortion strain energy density. Comput. Methods Appl. Mech. Eng..

[12] Zhao T., Ramos A.S., Paulino G.H. (2019). Material nonlinear topology optimization considering the von Mises criterion through an asymptotic approach: Max strain energy and max load factor formulations. Int. J. Numer. Methods Eng..

[13] Foster A.S.J., Gardner L., Wang Y. (2015). Practical strain-hardening material properties for use in deformation-based structural steel design. Thin Walled Struct..

[14] Li Z., Pasternak H. (2019). Experimental and numerical investigations of statistical size effect in S235JR steel structural elements. Constr. Build. Mater..

[15] Voyiadjis G.Z., Taqieddin Z.N. (2009). Elastic Plastic and Damage Model for Concrete Materials: Part I—Theoretical Formulation. Int. J. Struct. Chang. Solids.

[16] Lubliner J., Oliver J., Oller S., Oñate E. (1989). A plastic-damage model for concrete. Int. J. Solids Struct..

[17] Polus Ł., Szumigała M. (2019). Laboratory tests vs. FE analysis of concrete cylinders subjected to compression. AIP Conf. Proc..

[18] Jankowiak T., Lodygowski T. (2005). Identification of parameters of concrete damage plasticity constitutive model. Found. Civ. Environ..

[19] Gajewski T., Garbowski T. (2014). Calibration of concrete parameters based on digital image correlation and inverse analysis. Arch. Civ. Mech. Eng..

[20] Budziak M.P., Garbowski T. (2014). Failure Assessment of Steel-Concrete Composite Column Under Blast Loading. Eng. Trans..

[21] Liu Y., Gannon L. (2009). Experimental behavior and strength of steel beams strengthened while under load. J. Constr. Steel Res..

[22] Liu Y., Gannon L. (2009). Finite element study of steel beams reinforced while under load. Eng. Struct..

[23] Wang Y.Q., Zong L., Zhu R.X., Liu X.Y., Shi Y.J. (2015). Behavior of I-section steel beam welding reinforced while under load. J. Constr. Steel Res..

[24] Vild M., Bajer M. (2016). Strengthening under Load: The Effect of Preload Magnitudes. Procedia Eng..

[25] Al Ali M., Kvočák V., Platko P. (2017). Stress State of Steel Column Strengthened under Load. Procedia Eng..

[26] Pasternak H., Kubieniec G. (2016). Implementation of longitudinal welding stresses into structural calculation of steel structures. J. Civ. Eng. Manag..

[27] Deng D., Kiyoshima S. (2012). Numerical simulation of welding temperature field, residual stress and deformation induced by electro slag welding. Comput. Mater. Sci..

[28] Perić M., Garašić I., Nižetić S., Dedić-Jandrek H. (2018). Numerical Analysis of Longitudinal Residual Stresses and Deflections in a T-joint Welded Structure Using a Local Preheating Technique. Energies.

[29] Liu H., Zhao Y., Chen Z., Dong X. (2021). Axial-compression mechanical properties of square steel columns strengthened by welding under high load. J. Constr. Steel Res..

[30] Liu H., Hu J., Yang Y., Chen Z., Wang L. (2021). Circular steel tubes strengthened by welding angle steel under preloading condition. J. Constr. Steel Res..

[31] Szewczyk P., Szumigała M. (2016). Welding deformation in a structure strengthened under load in an empirical-numerical study. Proceedings of the Advances in Mechanics: Theoretical, Computational and Interdisciplinary Issues—3rd Polish Congress of Mechanics (PCM) and 21st International Conference on Computer Methods in Mechanics (CMM).

[32] Garsteckl A. (1984). Optimal Redesign of Elastic Structures in the State of Initial Loading. J. Struct. Mech..

[33] Garstecki A., Glema A. (1991). Sensitivity analysis and optimal redesign of columns in the state of initial distortions and prestress. Struct. Optim..

[34] Szewczyk P., Szumigała M. (2021). Strengthening of steel-concrete composite beams in experimental study. Mod. Trends Res. Steel Alum. Compos. Struct..

